# Editorial: Forest tree proteomics

**DOI:** 10.3389/fpls.2023.1285875

**Published:** 2023-09-26

**Authors:** Tiago S. Balbuena, María Ángeles Castillejo, Jesús Pascual

**Affiliations:** ^1^Department of Agricultural, Livestock and Environmental Biotechnology, School of Agriculture and Veterinary Sciences, São Paulo State University (UNESP), Jaboticabal, São Paulo, Brazil; ^2^Agroforestry and Plant Biochemistry, Proteomics and Systems Biology, Department of Biochemistry and Molecular Biology, University of Cordoba, Cordoba, Spain; ^3^Plant Physiology, Department of Organisms and Systems Biology, University of Oviedo, Oviedo, Spain; ^4^University Institute of Biotechnology of Asturias, University of Oviedo, Oviedo, Spain

**Keywords:** forestry, tree, proteomics, mass spectrometry, multi-omics

Covering more than 4.06 billion hectares around the world, forests harbor much of the terrestrial biodiversity (https://www.fao.org/state-of-forests/en/). They are important providers of ecosystem services and play a major role in climate change mitigation strategies due to their capacity to naturally remove large amounts of atmospheric CO_2_ (Hurteau, 2021). Their economic and ecological relevance is thus self-evident. Forests face major challenges, including an increasing demand for raw materials and climate change threats in the form of extreme climate events and the spread of aggressive pathogens (Hartmann et al., 2022). Tackling these challenges requires a deep understanding of the physiology of forest tree species. This Research Topic provides an overview of the current research in the field of forest tree proteomics, showing the relevance and power of research approaches used in this field to answer questions aimed at tackling ecological and economic challenges of the first magnitude for the course of our society. It is noteworthy that the papers included in this topic highlight the impact of an increasing amount of high-quality molecular information available in public repositories, which boosts the power of proteomic approaches, and the completion of the transition from gel-based to mass spectrometry-based methods that has taken place in the last decade. Furthermore, this Research Topic shows the increasing preponderance and power of multi-omics approaches.

The paper by Fan et al. represents a powerful approach in the field of proteomics. This approach consists of the comparison of different populations of the same species to gain new insights into a given process from the study of existing natural variation within the species. In this paper, the response to cold stress of two populations of *Camellia japonica*, one from a temperate geographical origin and one from a subtropical origin, was compared using a multi-omics approach. The combination of physiological, transcriptomic, proteomic, and hormonal dynamic analysis revealed the molecular basis of such a divergent stress resistance between the two studied populations. As hypothesized based on their geographical origin, the two populations showed a divergent resistance to the applied stress according to a collection of stress tolerance physiological markers. This approach allowed, along with the performance of co-expression analysis, to define modules of co-expressed genes in response to cold stress and revealed photosynthetic responses as the main cause for the contrasting stress tolerance. Furthermore, differences related to hormone signaling were identified and further validated by targeted hormone quantification. A core set of genes and proteins were equally responsive in both populations, which allowed for the defining of a conserved stress response inherent to the species regardless of stress tolerance natural variation in different populations.

Within the big topic of biotic stress, Cerny et al. aimed to explore the susceptibility of poplar to two aggressive pathogens of the oomycetes *Phythophthora* spp. responsible for agricultural losses and damage in natural ecosystems worldwide (Kamoun et al., 2015). As one of the most planted trees worldwide, poplars are an essential resource in forestry. No significant losses in the poplar population are caused by *Phytophthora.* However, the rapid growth of these trees requires a large water consumption, providing an optimal environment for pathogen spreading. As a fast-growing species, poplars are generally susceptible to stress (Piovesan and Biondi, 2021). Such inherent susceptibility hampers their mass cultivation and exploitation. In this context, there is a need to evaluate poplar species susceptibility to aggressive *Pythophtora* species. In their paper, Cerny et al. used a combined proteomic and metabolomic analysis to study the response to *P. cactorum* and *P. plurivora* in the *Pythophtora*-tolerant hybrid poplar clone T-14. Interestingly, their experimental design included sampling of wood microcores around the necrotic tissue at inoculation sites as well as from more distal zones, for protein spatial distribution analysis, and of leaves for the study of systemic responses. These analyses provide the first major insight into the molecular mechanisms of hybrid poplar defense against *Phytophtora*genus members. As a main result, a set of candidate tolerance markers is proposed.

Another important research field within forestry is the study of somatic embryogenesis (SE) for its potential biotechnological applications, especially the propagation of elite individuals or the *ex situ* conservation of endangered species (Guan et al., 2016). There is a great interest in understanding the determinants behind the capability of different cell lines to maturate into somatic embryos. Stress-related pathways are regarded as relevant in SE (Méndez-Hernández et al., 2019). The paper by Borges Araujo et al. explores the role of nitric oxide (NO), a main stress signaling component (Sánchez-Vicente et al., 2019), in SE. In particular, NO-induced cysteine S-nitrosilation during SE was studied in *Araucaria angustifolia*, an endangered South American pine species. The first S-nitrosoproteome of the SE process was defined in two cell lines selected for their contrasting embryogenic potential. Interestingly, proteins previously described as important in SE were identified as S-nitrosated. In addition, the changes in the endogenous levels of S-nitrosothiol and the S-nitrosoglutathione reductase activity at different SE stages were quantified. These results expand the knowledge on Brazilian pine SE and support NO signaling playing a significant role.

The contribution by Chacon et al. falls within the field of specialized metabolism, relevant as a source of bioactive compounds (Cragg and Newman, 2013). The authors reconstructed the flavonoid biosynthesis pathway in *Erythrina velutina*, a Brazilian native tree found in the unique semiarid biome of the Caatinga, using a multi-omics approach including proteomics, transcriptomics, and metabolomics. This tree species is a rich source of specialized metabolites, especially alkaloids and flavonoids, and it is widely used in traditional medicine (Fahmy et al., 2020). However, the identity of these metabolites and their biosynthesis pathways has remained mostly unexplored. Thus, the results by Chacon et al. provide the basis for further research aimed at identifying key regulators to achieve high metabolic fluxes toward the biosynthesis of compounds of interest.

Finally, Castillejo et al. provide an overview of the research concerning the genera *Pinus, Quercus*, and *Eucalyptus* during the last decade. A future perspective section covers the major advances to be seen in the coming years with special emphasis on the transition from single organism to population-wide studies and the bloom of proteomic-assisted molecular breeding.

## Author contributions

TB: Writing – review & editing. MC: Writing – review & editing. JP: Writing – original draft, Writing – review & editing.

## References

[B1] Cragg G. M. Newman D. J. (2013). Natural products: a continuing source of novel drug leads. Biochim. Biophys. Acta (BBA) General Subj. 1830, 3670–3695. doi: 10.1016/j.bbagen.2013.02.008 PMC3672862 23428572

[B2] Fahmy N. M. Al-Sayed E. El-Shazly M. Nasser Singab A. (2020). Alkaloids of genus Erythrina: An updated review. Nat. Prod. Res. 34, 1891–1912. doi: 10.1080/14786419.2018.1564300 31226894

[B3] Guan Y. Li S.-G. Fan X.-F. Su Z.-H. (2016). Application of somatic embryogenesis in woody plants. Front. Plant Sci. 7, 938. doi: 10.3389/fpls.2016.00938 27446166 PMC4919339

[B4] Hartmann H. Bastos A. Das A. J. Esquivel-Muelbert A. Hammond W. M. Martínez-Vilalta J.. (2022). Climate change risks to global forest health: emergence of unexpected events of elevated tree mortality worldwide. Annu. Rev. Plant Biol. 73, 673–702. doi: 10.1146/annurev-arplant-102820-012804 35231182

[B5] Hurteau M. D. (2021). “The role of forests in the carbon cycle and climate change,” in Climate change (Amsterdam, Netherlands: Elsevier), 561–579.

[B6] Kamoun S. Furzer O. Jones J. D. G. Judelson H. S. Ali G. S. Dalio R. J. D.. (2015). The Top 10 oomycete pathogens in molecular plant pathology. Mol. Plant Pathol. 16, 413–434. doi: 10.1111/mpp.12190 25178392 PMC6638381

[B7] Méndez-Hernández H. A. Ledezma-Rodríguez M. Avilez-Montalvo R. N. Juárez-Gómez Y. L. Skeete A. Avilez-Montalvo J.. (2019). Signaling overview of plant somatic embryogenesis. Front. Plant Sci. 10, 77. doi: 10.3389/fpls.2019.00077 30792725 PMC6375091

[B8] Piovesan G. Biondi F. (2021). On tree longevity. New Phytol. 231, 1318–1337. doi: 10.1111/nph.17148 33305422

[B9] Sánchez-Vicente I. Fernández-Espinosa M. G. Lorenzo O. (2019). Nitric oxide molecular targets: reprogramming plant development upon stress. J. Exp. Bot. 70, 4441–4460. doi: 10.1093/jxb/erz339 31327004 PMC6736187

